# Southeast Asia’s environmental challenges: emergence of new contaminants and advancements in testing methods

**DOI:** 10.3389/ftox.2024.1322386

**Published:** 2024-02-27

**Authors:** Jacky Bhagat, Nisha Singh, Yasuhito Shimada

**Affiliations:** ^1^ Graduate School of Regional Innovation Studies, Mie University, Tsu, Mie, Japan; ^2^ Mie University Zebrafish Research Center, Tsu, Mie, Japan; ^3^ Japan Agency for Marine-Earth Science and Technology (JAMSTEC), Yokosuka, Kanagawa, Japan; ^4^ Department of Bioinformatics, Mie University Advanced Science Research Promotion Center, Tsu, Mie, Japan; ^5^ Department of Integrative Pharmacology, Mie University Graduate School of Medicine, Tsu, Mie, Japan

**Keywords:** emerging contaminants, Southeast Asia, toxicity testing, environmental challenges, pollutants

## Abstract

Emerging contaminants, including pharmaceuticals, personal care products, microplastics, and per- and poly-fluoroalkyl substances, pose a major threat to both ecosystems and human health in Southeast Asia. As this region undergoes rapid industrialization and urbanization, the increasing presence of unconventional pollutants in water bodies, soil, and various organisms has become an alarming concern. This review comprehensively examines the environmental challenges posed by emerging contaminants in Southeast Asia and recent progress in toxicity testing methods. We discuss the diverse range of emerging contaminants found in Southeast Asia, shedding light on their causes and effects on ecosystems, and emphasize the need for robust toxicological testing methods. This review is a valuable resource for researchers, policymakers, and environmental practitioners working to mitigate the impacts of emerging contaminants and secure a sustainable future for Southeast Asia.

## Introduction

The burgeoning use of diverse chemicals in our everyday lives, ranging from pharmaceuticals and personal care products (PPCPs) to perfluorinated and polyfluorinated substances (PFAS), has raised concerns about their potential to contaminate the environment. Many substances went unnoticed in the past due to their low concentrations and a lack of information regarding their environmental accumulation. However, with advancements in analytical technology, these compounds have come into focus, challenging our conventional understanding of environmental pollution. Their widespread presence and potential to affect both human health and the environment have garnered significant scientific scrutiny to label them as “Emerging contaminants” (ECs).

ECs signify a shift in environmental research from studying single or grouped chemicals to understanding complex interactions among a wide range of chemicals that move through ecosystems. Unlike established contaminants with known harmful effects, ECs have not been detected using traditional monitoring methods. ECs are typically found in trace concentrations ranging from nanograms (ng) to micrograms (µg) per liter (Montagner et al., 2019; Lin et al., 2020; Selak et al., 2022). Over the past 2 decades, an exponential surge in the number of ECs discovered in natural environments has been reported (Noguera-Oviedo and Aga, 2016; Morin-Crini et al., 2022). In particular, pharmaceuticals (Valdez-Carrillo et al., 2020; Biswas and Vellanki, 2021), PPCPs (Akhbarizadeh et al., 2020; Lin et al., 2020), flame retardants (Du et al., 2019), and PFAS (Xiong et al., 2019), have been increasingly detected in the environment. A fraction of ECs is natural or transformed chemicals from synthetic materials potentially originating due to biogeochemical processes in the environment. Therefore, information on ECs is limited, and the methods to detect them are in the early stage of development.

ECs, including pharmaceuticals and engineered pesticides, are intentionally designed to be stable, durable, and resilient under challenging environmental conditions. The presence of ECs in environmental matrices, such as wastewater systems, is associated with human activities. Increasing numbers of ECs in the water bodies have often been reported in urban areas with high anthropogenic activities (Castiglioni et al., 2018; Biswas and Vellanki, 2021). Contaminants can infiltrate the environment through various routes, including wastewater discharge, agricultural runoff, waste dumping sites, industrial activities, urban runoff, and atmospheric deposition. The long-lasting presence of ECs in the environment raises concerns regarding their ability to accumulate in organisms and ultimately enter the human food chain.

The unprecedented ubiquity of chemicals in modern society necessitates a paradigm shift in toxicological evaluation, focusing not only on conventional compounds but also on the complex challenges posed by ECs (Meng et al., 2009). In addition to their widespread occurrence in matrices, the persistence of ECs has become a serious concern for ecotoxicologists and risk regulators (Krewski et al., 2010). ECs possess dynamic characteristics, are commonly found in very low concentrations in the natural environment, and have limited toxicity data available. The lack of established health standards for ECs underscores the urgent need for a comprehensive assessment of their presence, transport, and potential impact.

The rapid development, and population growth in Southeast Asia (Table 1) have led to a new era of pollutants that were previously not considered a major problem. In recent years, higher concentrations of ECs have been detected in different environmental matrices in this region (Boontanon et al., 2013b; Baluyot et al., 2021), including flame retardants such as decabromodiphenyl ethane, 1,2-bis-(2,4,6-tribromophenoxy)ethane, and dechlorane in sediments collected from e-waste (electronic waste) recycling sites in Vietnam (Someya et al., 2016). The CAS registry serves as a comprehensive repository of disclosed chemical substance data, encompassing over 204 million unique organic and inorganic substances and 70 million protein and DNA sequences. In contrast, the US EPA designates only 126 substances as priority pollutants under the Clean Water Act; this highlights the challenges that many environmental protection agencies in Southeast Asian countries face owing to limited resources and capacity (EPA, 2021).

**TABLE 1:** The distribution of emerging contaminants across Southeast Asian countries is influenced by several factors, including but not limited to population growth, urbanization trends, and agricultural practices, as demonstrated in the following table within each respective nation.

Countries	Total population (in 1,000)		Urbanization (in %)		Agricultural land area (in %)		Pesticides use in agricultural sector (in t)	
	2011	2021	2011	2021	2011	2021	2011	2021
Brunei	401.51	445.37	75.31	78.55	2.54	2.54	149.55	339.11
Cambodia	14,573.89	16,589.02	20.66	24.67	30.88	34.55	1,673	16,736.35
Indonesia	247,099.697	273,753.191	50.6	57.29	30.04	34.13	206,061.7	283,297.13
Laos	6,416.33	7,425.06	30.66	36.94	9.86	8.80	43.8	187.31
Malaysia	29,184.13	33,573.87	71.61	77.7	22.92	26.09	41,562.16	45,671
Myanmar	49,794.52	53,798.08	29.08	31.45	19.22	19.89	4,642.42	11,748.89
Philippines	96,337.91	113,880.33	45.52	47.68	41.12	42.54	21,179.54	37,660.36
Singapore	5,281.34	5,941.06	100	100	1.04	0.92	NA	NA
Thailand	68,712.85	71,601.10	44.7	52.16	44.68	46.00	87,191	19,006.56
Vietnam	88,349.12	97,468.03	31.08	38.05	34.37	39.43	30,302	50,096

**FIGURE 1:**
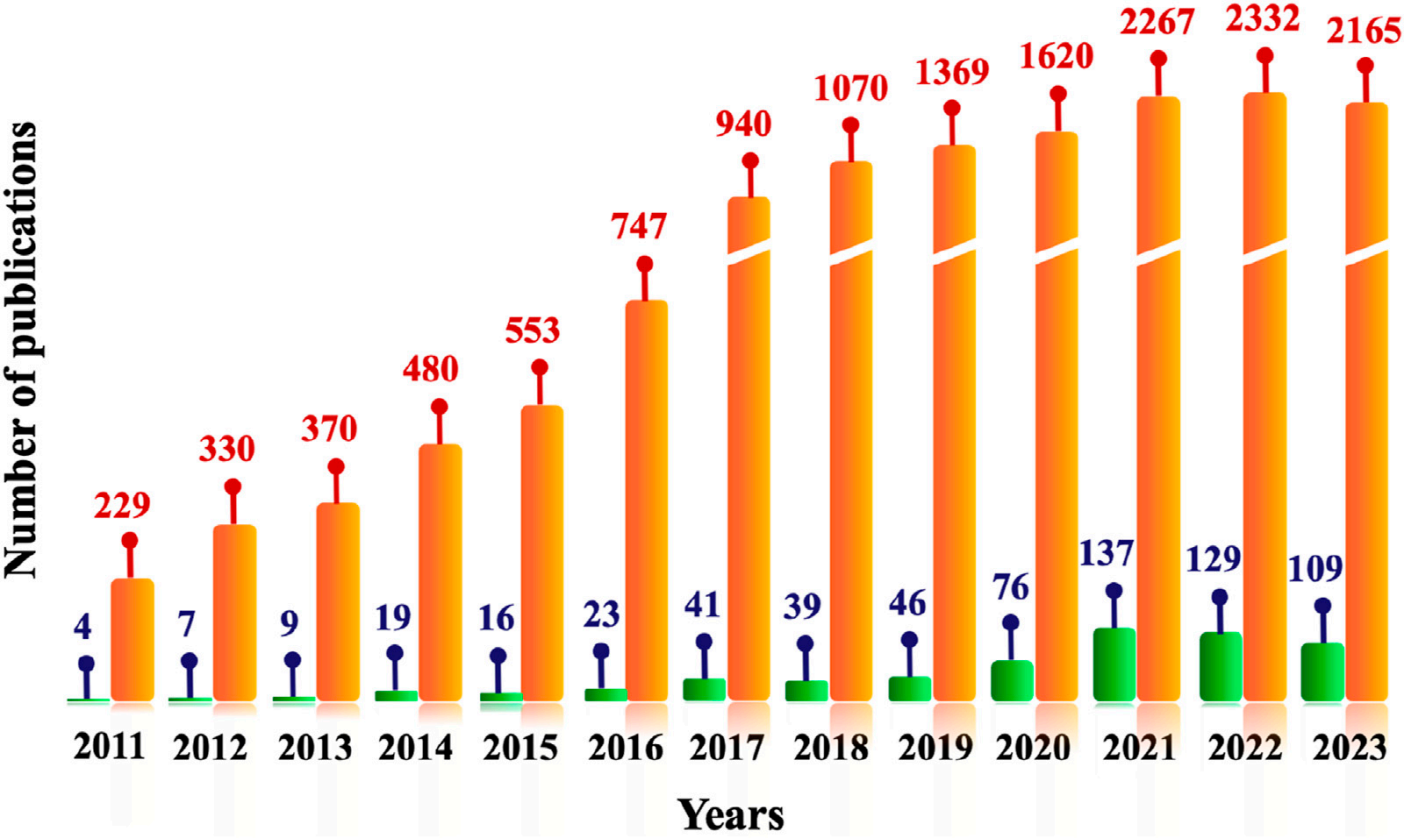
Number of manuscripts published on the topic of “emerging contaminants” worldwide (orange bar) and in Southeast Asian countries (green bar) from 2011 to 2023 (Source: Web of Science).

**FIGURE 2:**
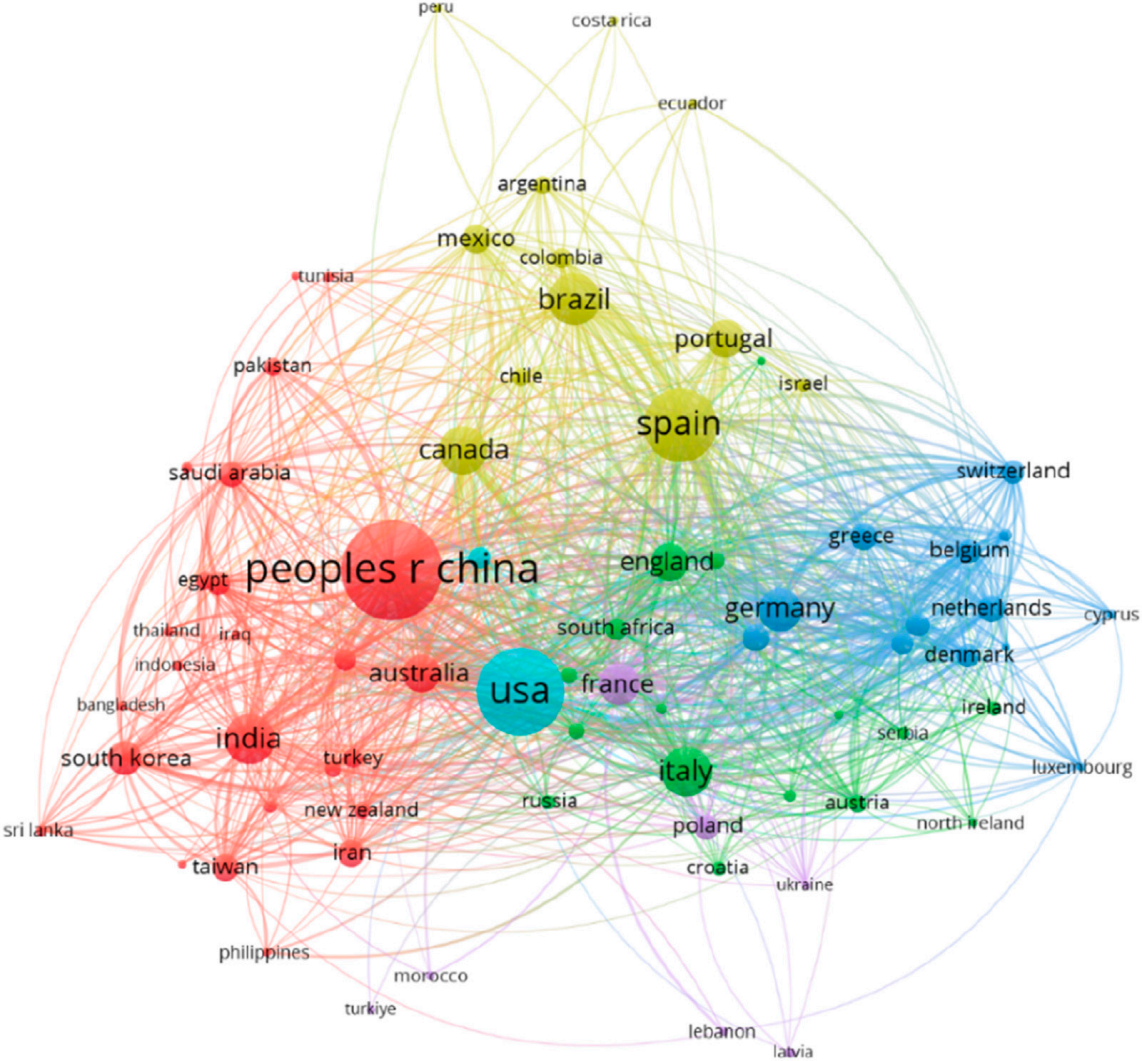
Country-wise publication trends of ECs from 2010 to 2023 in VOS viewer (https://www.vosviewer.com/).

Research publications on ECs have grown exponentially, resulting in a vast body of knowledge in North America, Europe, and parts of Asia (China and India) (Figures 1, 2). The rising number of review articles on ECs in Southeast Asia reflects a growing awareness of this issue in the region (Lam et al., 2017a; Menon et al., 2020; Chen et al., 2021). However, despite facing unique environmental challenges, comprehensive studies and reviews on ECs in Southeast Asia remain scarce. Without a comprehensive understanding of ECs in Southeast Asia, regulatory frameworks, policy decisions, and mitigation strategies remain incomplete. Considering these critical concerns, this review summarizes the latest research findings on the occurrence of ECs in Southeast Asian environments, their detection and testing approaches, challenges, and future directions of research.

## Challenges of emerging contaminants in Southeast Asian countries

**FIGURE 3:**
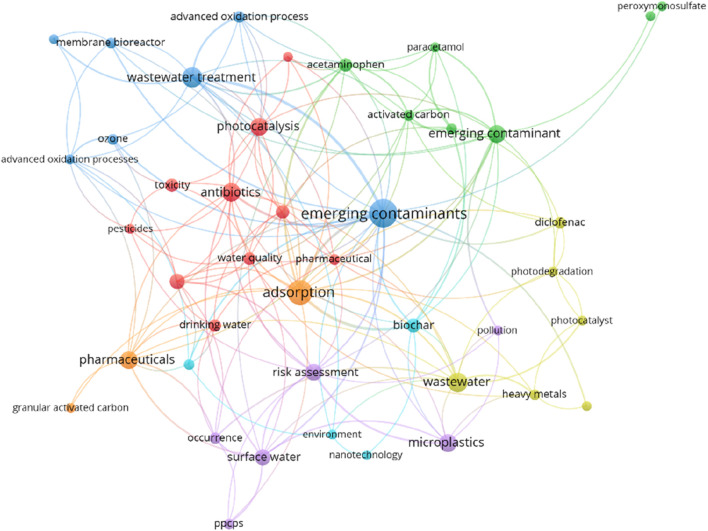
Keyword analysis of research papers from Southeast Asia from 2010 to 2023 on ECs using VOS Viewer.

The diverse countries of Southeast Asia are facing a growing number of ECs linked to rapid urbanization, industrialization, agricultural intensification, and lifestyle changes (Table 1). To visualize trends in EC research in Southeast Asia, we conducted a comprehensive keyword analysis of research papers published between 2010 and 2023. Figure 3 displays the most frequent keywords found in these papers. The concern of ECs in the region is linked to local factors, regulatory frameworks, socioeconomic dynamics, and environmental conditions (Abu Hasan et al., 2023). These issues vary by country. For example, Cambodia has seen a drastic ten-fold increase (Table 1) in the use of pesticides and agrochemicals with no significant change in the agricultural land area. Although vital for the country’s economy and food security, the over-application of pesticides raises concerns about water quality and aquatic ecosystem health in the region (Irvine et al., 2006; Meas et al., 2021). Additionally, it also introduces an important dimension to the discussion of ECs. The tropical climate and diverse agricultural systems in Cambodia compound the complexity of contamination pathways, emphasizing the urgency for rigorous research and targeted interventions in the region.

The rapid expansion of urban areas and increased use of agrochemicals in Indonesian agriculture (Table 1) contribute to the infiltration of chemicals into the environment. Further, marine and freshwater ecosystems in Indonesia are threatened by ubiquitous plastic waste and microplastics from inland and the surrounding countries via ocean currents (Vriend et al., 2021). After China’s waste import bans in 2018, statistics from the Badan Pusat Statistik revealed a staggering 141% surge in Indonesia’s plastic waste imports, amounting to 283,152 tons (Badan Pustak Statistik, 2018). In addition to microplastics, increasing aquaculture operations in this region have resulted in a significant increase in the presence of pharmaceuticals of emerging concern (Hidayati et al., 2021; Sudaryanto et al., 2023).

The palm oil industry in Malaysia is a major contributor to ECs concerns, via the use of pesticides and fertilizers, which can negatively impact soil and water quality (Igwe and Onyegbado, 2007). Malaysia’s position as a global hub for the electronics and automotive sector makes it vulnerable to ECs from industrial activities (Azad et al., 2017). Chemicals used in the electronics sector, such as flame retardants and plasticizers, are found in the environment and pose potential health and ecological risks. Urban centers in Malaysia, driven by population growth (Table 1), are a major source of environmental contaminants. Furthermore, PPCPs, and endocrine disruptors are often reported in Malaysian water streams (Farid et al., 2016; Hasni et al., 2023).

The Philippines is known for its rich biodiversity and diverse ecosystems. Being one of the world’s largest producers of pineapples, sugar, and coconut products, pesticides, herbicides, and fertilizers are used to boost agricultural output. The country’s topography and tropical climate intensify agricultural runoff, which introduces ECs into aquatic ecosystems, disrupting the delicate balance between freshwater and marine ecosystems (Lu, 2010; Lu et al., 2010). As the country’s socioeconomic landscape evolves with fast-growing urban centers, expanding population, and changing consumer habits, it faces a pressing environmental challenge from ECs such as polybrominated diphenyl ethers, pesticides, and artificial sweeteners (Watanabe et al., 2016; Hoang et al., 2021; Mohd Nizam et al., 2023).

Singapore’s urban environment, with limited land resources and a dependence on imported water, faces challenges in terms of water security due to ECs from urban effluents. The high urban regions warrant heightened PPCPs and microplastics in urban effluent that can escape the wastewater treatment plants due to inefficient removal thus compromising the national water security (Xu et al., 2011b; Bayen et al., 2016b). Some other well-known sources for ECs leakage into the environment are landfill sites, firefighting drills, and recreational activities.

In Thailand, rapid urbanization has contributed to the contamination of water bodies with pharmaceuticals, and personal healthcare products (Juksu et al., 2020; Chan et al., 2022). Incomplete removal of ECs from industrial wastewater treatment including textile, electronics, plastics, and chemicals a major source of ECs in the country. Significant quantities of synthetic musks and UV filters from urban settlements serve as key components of ECs. Synthetic musk has been reported in the coastal waters of Thailand (Juksu et al., 2020).

Vietnam has emerged as a major contributor to the global burden of ECs pollution due to electronic waste and quick urban growth. The country has become a hub for electronic waste recycling because of its low recycling costs and weak enforcement of environmental laws. The improper recycling of electronic waste in Vietnam and other South Asian countries has led to a significant increase in flame-retardant pollution (Lee et al., 2022).

## Emerging contaminants in the Southeast Asian environment

### Pharmaceuticals and personal care products

Amidst the myriad challenges faced by Southeast Asia, PPCPs contamination of aquatic ecosystems and water resources is a prominent concern. PPCPs constitute an expanding category of emerging organic microcontaminants that act as harmful agents and disturb biological and ecological systems. PPCPs pollution primarily originates from the release of untreated or poorly treated wastewater from hospitals, drug factories, and homes into rivers, lakes, and coastal waters.

Their widespread use and improper disposal have been reported in all types of water bodies in Southeast Asia, including surface waters (Tewari et al., 2013; Al-Qaim et al., 2015), rivers (Praveena et al., 2018; Da Le et al., 2021), lakes (Tran et al., 2019a; Duong et al., 2021a), and groundwater (Tran et al., 2014) (Table 2). PPCPs that are frequently identified in the environment include antibiotics, antipyretics, caffeine, hormones, synthetic musks, and insect repellents. These substances can enter the environment through improper disposal, excretion, or even by washing off our bodies and draining. Upon entering aquatic ecosystems, PPCPs bind to organic substances, undergo decomposition via physical and chemical mechanisms, and accumulate within the food chain. PPCPs adversely affect aquatic life and human health. A cross-country study in Asia highlighted the bioaccumulation of PPCPs in tilapia, carp, and catfish collected from Indonesia and Vietnam (Nozaki et al., 2023).

**TABLE 2:** Concentration of emerging contaminants in different environmental matrices in Southeast Asia.

EC category	EC name	Matrix	Location	Concentration range	References
PPCPs	N,N-diethyl-3-toluamide (DEET), Ibuprofen, O-desmethyl tramadol, Mefenamic acid, Sulfamethoxazole and Tramadol	Seawater	Indonesia	n.d.–170 ng/L	Sudaryanto et al. (2023)
PPCPs	Caffeine, acetaminophen, *N,N-diethyl-m*-toluamide (DEET), ibuprofen (IBU), and triclosan	Municipal wastewater	Indonesia	29.8 ± 0.4 μg/L	Astuti et al. (2023)
PPCPs	Cyclic volatile methyl siloxanes	Indoor dust	Vietnam	86.0–5,890 ng/g	Hoang et al. (2022)
PPCPs	Butyl methoxydibenzoylmethane, benzophenone-3, acetaminophen, cotinine, and fuorescent brightener	Air sample	Vietnam	0.61–21.9 ng m−3	Duong et al. (2023)
PPCPs	Carbamazepine, sulfamethoxazole, erythromycin, and triclosan	Plasma of fish	Indonesia, Vietnam	n.d.–110 ng/mL	Nozaki et al. (2023)
PPCPs	Acetaminophen, caffeine, sulfamethazine, metformin, iopamidol, sulfamethoxazole, acetylsulfamethoxazole, ciprofloxacin, and azithromycin	Freshwater samples	Philippines	1848.57 ng/L	Mariano et al. (2023)
PPCPs	Biocide Irgarol-1051	Coastal water	Malaysia	2021 ng/L	Ali et al. (2013)
PPCPs	Diclofenac, BPA, progesterone, and amoxicillin	Fish and mollusks	Malaysia	0.73–10.76 ng/g	Omar et al. (2019a)
PPCPs	Pharmaceutically active compounds and endocrine disrupting chemicals	Mangrove water sediment mollusks	Singapore	366 ng/L 81 ng/g dw 33 ng/g ww	Bayen et al. (2016a)
PPCPs	Sulfonamides, trimethoprim, macrolides, lincomycin, tetracyclines	Water sample	Indonesia, Vietnam, Malaysia, Philippines	282 ng/L 1720 ng/L 76 ng/L 802 ng/L	Shimizu et al. (2013)
PPCPs	Artificial sweeteners	River and wastewater	Vietnam, Philippines, Myanmar	2,600 ng/L	Watanabe et al. (2016)
PPCPs	PPCPs	Wastewater	Vietnam	7.6 μg/L	Nguyen et al. (2018)
PPCPs	Acetaminophen, acetylsalicylic acid, atenolol, caffeine, ciprofloxacin, diclofenac, ibuprofen, mefenamic acid, naproxen, roxithromycin, sulfamethazine, sulfamethoxazole, sulfathiazole and trimethoprim	Wastewater Surface water	Thailand	1–16,000 ng/L 313 ng/L	Tewari et al. (2013)
PPCPs	Macrolides, sulfonamides, β- lactams, lincomycin, chloramphenicol, furazolidon, and monensin	Surface water Soil	Singapore	82.5 ng/L 80.6 ng/g dw	Yi et al. (2019)
PPCPs	Caffeine, prazosin, enalapril, carbamazepine, nifedipine, gliclazide, levonorgestrel, simvastatin, hydrochlorothiazide, diclofenac-Na and mefenamic acid	Surface water	Malaysia	20–1,644 ng/L	Al-Qaim et al. (2015)
PPCPs	Synthetic musk fragrance compounds, methyl triclosan	Mangrove sediment	Singapore	n.d.-670 ± 216 ng/g	Bayen et al. (2019)
PPCPs	Antibiotics	Urban canals	Vietnam	n.d. – 50,000 ng/L	Tran et al. (2019a)
PPCPs	Amoxicillin, caffeine, chloramphenicol, ciprofloxacin, dexamethasone, diclofenac, nitrofurazone, sulfamethoxazole, and triclosan	Drinking water	Malaysia	0.14–0.38 ng/L	Praveena et al. (2019a)
PPCPs	Organic micro-pollutants	River sediment	Vietnam	1,600–247,000 ng/g d.w.	Duong et al. (2014)
PPCPs	PPCPs	Fish and mollusk	Malaysia	0.73–10.76 ng/g	Omar et al. (2019b)
PPCPs	Pharmaceutically active compounds	Surface water	Malaysia	102.31 ng/L	Omar et al. (2018)
PPCPs	Active pharmaceutical ingredients	Drinking Water	Malaysia	21.39 ng/L	Wee et al. (2020)
PPCPs	Synthetic musks, triclosan and methyl triclosan	Water and sediment	Singapore	0.08–6.45 ng/L 0.082–5.5 ng/g d.w.	Wang and Kelly (2017a)
PPCPs	Atenolol, acetaminophen, theophylline, caffeine, metoprolol, and sulfamethoxazole	Surface water, sewage treatment plant, and hospital samples	Malaysia	8,700 ng/L	Al-Qaim et al. (2018)
PPCPs	Antibiotics and progesterone	Broiler manure And agricultural soil	Malaysia	78,516 μg/kg d.w.	Ho et al. (2014)
PPCPs	Synthetic musks and benzotriazole UV stabilizers	Mussels	Indonesia, Malaysia, Philippines, Vietnam	3,300 ng/g lipid wt.	Nakata et al. (2012)
PPCPs	Alkylphenol ethoxylate metabolites (APEMS), hormones, pharmaceuticals, bisphenol A, and a pesticide (FIPRONIL)	Surface water	Singapore	n.d. – 6 μg/L	Xu et al., 2011a
PPCPs	Organic acids (oxalate, succinate, adipate, maleate, phthalate) and methanesulfonate (MSA)		Manila, Philippines	149 ± 94 ng/m3	Stahl et al. (2020)
PPCPs, EDC	Inorganic and organic contaminants	Floodwater in paddy field	Vietnam	3,170 μg/L	Trinh et al. (2017)
PPCPs, EDC	Organic micro-pollutants	Ground water	Vietnam	16 μg/L	Duong et al. (2015a)
PPCPs, EDC	Organic micropollutants	Coastal sediment	Indonesia	1,615 ng/g d.w.	Koike et al. (2012)
PPCPs, EDC	Organic micropollutants	Surface water	Vietnam	194 μg/L	Chau et al. (2018)
PPCPs, EDC	Organic chemical pollutants	Lake water	Philippines	n.d. – 1,150 ng/L	Dimzon et al. (2018)
EDC	17α ethinylestradiol, levonorgestrel, norethindrone and cyproterone acetate	River water	Malaysia	n.d. – 1898 ng/L	Al-Odaini et al. (2013)
EDC	Endocrine-disrupting compounds	Estuarine water	Malaysia	0.47–79.89 ng/L	Ismail et al. (2019a)
EDC	Lipophilic organic contaminants	Surface water	Indonesia	23,900 ng/L	Dsikowitzky et al. (2016)
EDC	Bisphenol A and alkylphenols	Fish	Malaysia	0.023–0.322 ng/g	Ismail et al. (2018)
EDC	Estrogenic chemicals	River water	Malaysia	3.6–37 μg/L	Nazifa et al. (2020)
EDC	BPA	Surface water, drinking water, blood serum	Malaysia	215 ng/L 3.3 ± 2.6 ng/L 0.81–3.65 ng/mL	Santhi et al. (2012)
FR	Polychlorinated biphenyls (PCBs), phthalate esters (PAES) and polybrominated diphenyl ethers (PBDES)	Surface water	Vietnam	1.89–412.27 ng/L	Quynh and Toan (2019)
FR	PBDES	Surface water	Vietnam	63 ng/g dw	Li et al. (2016)
FR	PBDE, dechlorane plus (DP), decabromodiphenylethane (DBDPE), hexabromocyclododecane (HBCD), hexabromobenzene (HBB), pentabromoethylbenzene (PBEB), pentabromobenzene, (PBBZ), and tetrabromo-p-xylene (PTBX)	Tree bark	Indonesia	46.1 ng/g Lipid Weight	Salamova and Hites (2013)
FR	Organophosphorus flame retardants (PFRs) and phthalates	Floor and road dust	Thailand	36–1,700 ng/g d.w. 86,000–790,000 ng/g d.w.	Muenhor et al. (2018)
FR	Phthalate Esters	Surface water	Thailand	1.44–12.08 μg/L	Kingsley and Witthayawirasak (2020b)
FR	Phthalate Esters	Sediment	Thailand	190–2010 ng/g d.w.	Kingsley and Witthayawirasak (2020a)
FR	Phthalate esters	Water and sediment	Thailand	0.23–0.91 μg/L n.d. – 1.65 μg/g	Malem et al. (2019)
FR	Halogenated flame retardants (HFRS) and personal care products (triclosan and synthetic musks	Leachate and water	Singapore	22.4 ng/L	Wang and Kelly (2017b)
FR	Halogenated flame retardants	Marine sediment	Singapore		Zhang and Kelly (2018)
FR	Polychlorinated biphenyls (PCBS), and additive brominated flame retardants (BFRS)	Indoor dust	Vietnam	5.4–1,400 ng/g	Tue et al. (2013)
FR	Polybrominated diphenyl ethers (PBDES)	Sediment	Vietnam	1.1–26 ng/g dry weight	Hoang et al. (2023a)
FR	PBDEs and HBCDs	Sediment	Indonesia	n.d. – 420 ng/g dw	Ilyas et al. (2011)
FR	Polybrominated diphenyl ethers (PBDES)	Dust from urban and rural areas	Vietnam	36–650 ng/g	Hoang et al. (2023b)
FR	Phthalic acid esters	Bottled water, tap water, lake water, and wastewater	Vietnam	1,640–405,000 ng/L	Le et al. (2021)
Pesticides	Pesticides	Chinese kale	Thailand	62,500 μg/kg	Wanwimolruk et al. (2015)
Pesticides	Biocides	Wastewater and sludge	Thailand	15.2 μg/L 8.47 μg/g	Juksu et al. (2019)
Pesticides	DDT and its metabolites	Source water	Malaysia	0.6–14.6 ng/L	Veerasingam and Ali (2013)
Microplastics	Microplastics	Soil	Cambodia Indonesia Laos Philippines Vietnam	26,749 ± 67,488 pieces/kg d.w. 10,929 ± 13,547 pieces/kg d.w 8,402 ± 7,872 pieces/kg d.w 20,333 ± 12,897 pieces/kg d.w 20,608 ± 23,633 pieces/kg d.w	Tun et al. (2022)
Perfluorinated compounds (PFAS)	Perfluorohaptanoic acid (pfhpa), perfluorooctanoic acid (PFOA), perfluorononanoic acid (PFNA), perfluorodecanoic acid (PFDA), perfluoroundecanoic acid (PFUNA), perfluorohexane sulfonate (PFHXS), and perfluorooctane sulfonate (PFOS)	Ground water	Thailand	1.68–42.01 ng/L	Hongkachok et al. (2018)
PFAS	Trifluoromethanesulfonic acid (TFMS), pentafluoropropionic acid (PFPRA), trifluoroacetic acid (TFA), 1-perfluopropylethanol, tridecafluoro-2-(trifluoromethyl) octanoic acid	Drinking water and source water	Philippines, Thailand	0.02–8.43 ng/L 0.04–90.17 ng/L	Guardian et al. (2020)
PFAS	Perfluorooctanoic Acid (PFOA) and Perfluorooctane Sulfonate (PFOS)	Surface water	Malaysia	n.d. – 43.5 ng/L	Zainuddin et al. (2012)
PFAS	PFOS and PFOA	Seafood River water Sea	Thailand	29–6,724 ng/kg w.w. 0.60–465.65 ng/L < 0.25–59.29 ng/L	Lertassavakorn et al. (2021)
PFAS	PFAS	Serum of breast cancer patients	Philippine	0.04–23.03 ng/mL	Velarde et al. (2022)
PFAS	PFOS and PFOA	Surface water	Singapore	n.d. – 156 ng/L	Nguyen et al. (2011)
PPCPs,FR	n-alkanes, PAHs, phthalates, and PPCPs	Road dust	Vietnam	7.8–170 μg/g	Anh et al. (2019)
PFAS	PFCs	Dust	Thailand		Goosey and Harrad (2011)
PFAS, EDC	Perfuoroalkyl and polyfuoroalkyl substances, bisphenols, and parabens	Tap water	Malaysia	0.28–5,516 ng/L	Haron et al. (2023)
PFAS	PFHXA, PFOA, PFBS and PFOS	Wastewater	Vietnam	7.99 ng/L	Nguyen (2023)
PFAS	Perfuorooctanoic acid and perfuorooctane sulfonate	Ground water	Thailand	n.d.–34.96 ng/L	Hongkachok et al. (2023)
EDCs	Nonylphenols, DEET (N,N-diethyl-m-toluamide), DDT	Sediment	Indonesia	370 ng/g	Dsikowitzky et al. (2020)
PFAS	PFCs	Waters, sediment and biota (crustacean, gastropod, fish, bivalve)	Vietnam	53.5 ng/L, 16.9 ng/g w.w.	Lam et al. (2017a)
PFAS	PFCs	Leachate, surface water and sediment	Vietnam	3.81–328 ng/L, 3.52–6.70 ng/g	Ngoc et al. (2021)
PFAS	Perfluorooctanoic acid (PFOA), perfluorononanoic acid (PFNA), and Perfluoroundecanoic acid (PFUDA)	Water	Vietnam	0.5–360 ng/L	Kim et al. (2013)
PFAS	Perfluoroalkyl acids	Ground water and river water	Vietnam	0.45–18 ng/L	Duong et al. (2015b)
PFAS	PFOS	Water sample	Thailand	100.8 ng/L	Boontanon et al. (2013a)
PFAS	PFAS	Surface water	Singapore	520 ng/L	Chen et al. (2017)
PFAS	PFCs	Water and sediment	Singapore	11–253 ng/L 8–42 ng/g	Nguyen et al. (2016)
PFAS	PFOS and PFOA	Wastewater and sludge	Singapore	31.5 ng/L 29.8 ng/g	Yu et al. (2009)
PFAS	PFCs	Sediment	Singapore	4,700 ng/kg	Nguyen et al. (2012)
PFAS	Perfluorinated surfactants	Freshwater fish	Vietnam, Malaysia, Thailand	0.05–0.3 ng/g w.w.	Murakami et al. (2011)
PFAS	Perfluoroalkyl acids	Blood serum	Malaysia	32.57 ng/mL	Ramli et al. (2020)
UV filters	Benzophenone-1 (BP-1), benzophenone-3 (BP-3), benzophenone-4 (BP-4), benzophenone-8 (BP-8), ethylhexyl salicylate (EHS), isoamyl p-methoxycinnamate (IAMC), octyl dimethyl-p-aminobenzoic acid (ODPABA), butyl methoxydibenzoylmethane (BMDM), ethylhexyl methoxycinnamate (EHMC), homosalate (HMS), 4-methylbenzylidene camphor (4-MBC), and octocrylene (OC)	Surface water	Thailand	28–205 ng/L	Tsui et al. (2014)

Fragrances, whether synthetic or natural, are a category of additives commonly used in personal care products and household goods. In a study of a tropical urban catchment in Singapore, synthetic musks, triclosan, and methyl triclosan were found to be widespread in both the dissolved and solid phases (Wang and Kelly, 2017a). Fragrances, including synthetic musks, can have adverse effects on aquatic ecosystems (Luo et al., 2023). Artificial sweeteners, such as saccharin and sucralose, are extensively used in various food, pharmaceutical, and personal hygiene products. These compounds often enter domestic wastewater systems (Watanabe et al., 2016). The use of veterinary drugs in livestock, improper disposal of unused medicines, and feeding of animals with ingredients containing pharmaceuticals are major sources of pharmaceutical pollution in agricultural fields. Jarat et al. (2018) the agricultural fields near a swine farm in Thailand were contaminated with antibiotics. Studies in Thailand and Malaysia have identified antibiotics, analgesics, anti-inflammatory drugs, and antiepileptic drugs in rivers, surface water, and drinking water, suggesting that pharmaceutical pollution is more widespread than previously thought (Praveena et al., 2019b; Khan et al., 2020). Similarly, studies in Hanoi and Manila have identified antibiotics, antiparasitic residues, and stimulants (caffeine) in urban rivers (Da Le et al., 2021; Van et al., 2021). A detailed study of 21 rivers and canals in Vietnam reported the presence of 950 organic micropollutants, including caffeine, pesticides, and PPCPs (Hanh et al., 2014).

### Endocrine-disrupting compounds

Southeast Asia has been facing a hidden but severe challenge of EDCs contamination. Studies conducted in Southeast Asia revealed the widespread presence of EDCs in the Mahakam River in Indonesia (Hadibarata et al., 2020), Malaysia (Ismail et al., 2019b; Ismail et al., 2020), Singapore (Bayen et al., 2013; You et al., 2015), and Vietnam (Quynh and Toan, 2019). Octylphenol, nonylphenol, bisphenol-A (BPA), and estrone are EDCs commonly used in agriculture and household products and have been reported in water, sediments, and fish collected from the Nan River, Thailand (Deemoon et al., 2016). The discharge of partially treated municipal effluents into water bodies is a major source of EDCs contamination in this region. As part of the mussel watch program, an extensive study conducted by Isobe et al. (2007) at 48 locations in Asia showed contamination of octylphenol, nonylphenol, BPA, and linear alkylbenzenes in green mussels (*Perna viridis*). Another investigation by Ocharoen et al. (2018) reported high levels of BPA and 17β-estradiol in *P. viridis* correlated with contamination in collection sites. Industrial areas in Southeast Asia are hotspots for EDCs, and studies have shown that discharge from industrial processes is a major source of contamination (Am Hung Viet et al., 2005).

### Per- and poly-fluoroalkyl substances

PFAS are fluorinated chemicals, which are resistant to heat, water, and grease, and are used in a wide range of industrial and consumer products, such as non-stick cookware, water-repellent textiles, electronics manufacturing, and food processing industries and PFAS-containing aqueous film-forming firefighting foams. PFAS features numerous carbon-fluorine bonds, endowing with thermochemical stability and remarkable resistance to degradation. Perfluorooctanoic acid (PFOA) and perfluorooctane sulfonate (PFOS) are extensively used members of more than 4,000 compounds in the PFAS family, across diverse industries (Glüge et al., 2020; Brennan et al., 2021). Despite global efforts to phase out PFAS under the Stockholm Convention on Persistent Organic Chemicals, they endure in the environment as “forever chemicals.” PFAS structural characteristics, encompassing factors such as functionality, carbon-chain length, hydrophobicity, and lipophobicity govern their fate in the environment. The International Agency for Research on Cancer (IARC) has designated PFOA as a substance that is ‘possibly carcinogenic to humans’ (Zahm et al., 2024).

PFAS have infiltrated Southeast Asia’s air, water, soil, and living organisms, raising concerns about their impact on ecosystems and human health. Recently, several studies and reviews have been published on the occurrence of PFAS in Southeast Asian environments (Baluyot et al., 2021; Singh et al., 2023; Tang et al., 2023). Moreover, PFAS-containing aqueous film-forming foams used in emergencies have been released into aquatic environments. PFOA and PFOS are the most studied PFAS in Southeast Asia, with most publications from Vietnam, Thailand, and Singapore (Lee et al., 2022). Studies detailed in Table 2 demonstrate the presence of PFAS in Singapore’s surface water to a level of 156 ng/L (Nguyen et al., 2011).

A nationwide survey of aquatic environments in Vietnam has documented the highest recorded concentrations of PFOA and PFOS, with levels reaching 53.5 ng L^−1^ and 40.2 ng L^−1^, respectively (Lam et al., 2017a). Guardian et al. (2020) showed that PFAS prevalence is widespread in the Philippines and Thailand, even in drinking and source waters. Although the PFAS concentrations were below the US EPA limit (70 ng/L), this study highlighted the need for frequent monitoring programs at refill stations. PFAS concentrations as high as 43.5 ng/mL have been reported in surface waters in Malaysia (Zainuddin et al., 2012). Research by Hongkachok et al. (2023) on PFAS continuation in groundwater highlighted the risk of leaching into drinking water, which poses a threat to human health and ecosystems.

### Microplastics

Microplastics, tiny plastic pieces <5 mm, are found everywhere in nature and can harm the environment and human health. Southeast Asia, a hotspot for plastic production and consumption, generates vast amounts of plastic waste that can fragment into microplastics and pollute its waterways. Jambeck et al. (2015) reported that plastic waste in Indonesia, Malaysia, the Philippines, Thailand, and Vietnam, which account for approximately 7.5% of global pollution, collectively contributed to approximately one-third of the world’s marine plastic pollution. Limited waste management infrastructure, informal recycling practices, and prevalent aquaculture and fishing have raised concerns about human exposure to microplastics through seafood consumption. The diverse economies in this region drive the proliferation and use of plastic products through industrial growth and consumer product manufacturing. An assessment by Meijer et al. (2021) indicated that 29 rivers of the top 50 global contributors to plastic emissions are from Southeast Asia.

Chen et al. (2021) published a comprehensive review of microplastics and identified their sources and transport pathways in freshwater ecosystems in Southeast Asian countries. Recent research has revealed the alarming presence of microplastics in river ecosystems in Southeast Asia, highlighting widespread contamination by tiny plastic particles (Babel et al., 2022). Ng et al. (2023) found plastic waste and microplastics in a variety of environmental compartments, including marine waters, freshwater systems, soils, and sediments across multiple Southeast Asian countries and highlighted that countries with higher population densities along their coastlines, such as Indonesia, the Philippines, and Vietnam, are more likely to pollute the ocean with plastic.

Recent studies have also identified microplastics in terrestrial ecosystems and agricultural landscapes in Southeast Asia. For example, Doan et al. (2023) found microplastics in the agricultural soils of Thailand and warned that they could harm crops. Tran-Nguyen et al. (2022) examined the extent of microplastic pollution in a drainage channel in Da Nang, Vietnam, and reported concentrations as high as 1,482.0 ± 1,060.4 items m^−3^ in waters and 6,120.0 ± 2,145.7 items kg^−1^ in sediments. Sin et al. (Sin et al., 2023) indicated that water-based environments, such as rivers, estuaries, beaches, and seas, are the primary sites of substantial contamination. Microplastics readily enter these aquatic habitats, facilitated by air movement and rainfall patterns. In a recent study, New et al. (2023) investigated the levels of microplastics in drinking water across Southeast Asian countries and highlighted the potential risks and health implications.

### Flame retardants

Flame retardants are ubiquitous in everyday products ranging from electronics and furniture to textiles and construction materials. In recent decades, Southeast Asia’s rapid industrialization, urbanization, and economic growth have made the consequences of flame-retardant pollution increasingly evident. In recent years, Vietnam has become a hub for electronic manufacturing and e-waste recycling, increasing the demand for flame retardants. An estimated 80% of electronic waste (e-waste) originating from industrialized nations is directed toward developing Asian countries for recycling, capitalizing on low labor costs and lax enforcement of environmental regulations (Network, 2002). Owing to the lack of strict regulations on flame retardants, these compounds are found in many consumer and commercial products and waste in Southeast Asia. In an extensive study, fishmeal collected from Thailand, Vietnam, and Malaysia was shown to contain brominated flame retardants (Li et al., 2019). A recent review highlighted Vietnam’s e-waste management practices, environmental impacts, and human exposure risks in informal recycling areas (Hoang et al., 2023c). Accumulation of hexabromocyclododecanes in human breast milk has been reported in the inhabitants of Vietnam e-waste sites (Tue et al., 2010).

Flame retardants that persist in the air can travel long distances and contribute to water pollution. Researchers have reported the presence of flame retardants in the surface waters of Indonesia (Dsikowitzky et al., 2016), soils and river sediments from Vietnam (Matsukami et al., 2012; Someya et al., 2016), and floor and road dust collected from Thailand (Muenhor et al., 2010). In the study (Muenhor et al., 2018), researchers have reported concentrations as high as 2,700 μg/g in floor dust from e-waste dismantling facilities in Thailand. Singapore’s global business and technology hub status drives the high consumption of flame-retardant products, which are essential for fire safety. The presence of halogenated flame retardants has been reported in urban watersheds (Bayen et al., 2003) and *P. viridis* (Hoang et al., 2023c) in Singapore.

### Pesticides and herbicides

The global pesticide production has undergone a twofold increase since 1990, with Southeast Asia experiencing a significant surge. This growth is propelled by the region’s expanding population and thriving agricultural exports. Recent studies on the presence of pesticides in aquatic environments have raised concerns regarding its widespread contamination in surface waters, rivers, and lakes (Duong HT. et al., 2021; Mohd Nizam et al., 2023). These investigations have highlighted the ubiquity of pesticides in this region, including organophosphorus pesticides (Giger et al., 2003; Wee et al., 2016), organochlorine pesticides (Tran TAM. et al., 2019), and carbamates (Tongpoo et al., 2015).

Several Southeast Asian countries have grappled with pesticide pollution, each with its unique challenges and environmental consequences. As an agriculture-based economy, Vietnam’s import of pesticides has grown exponentially in recent decades, both in terms of quantity and value (Van Hoi et al., 2013). Smallholder farmers dominate the agricultural sector in Southeast Asian countries. An interesting study conducted on small-scale farmers from Cambodia, Laos, and Vietnam reported that farmers who consulted friends and neighbors experienced a 45% reduction in pesticide usage (Schreinemachers et al., 2017). In contrast, those who sought guidance from pesticide retailers exhibited a significant increase of 251% in their pesticide usage. Furthermore, the study also stated that when women were in charge of pest management, pesticide use decreased by 42%, and the adoption of biopesticides led to a 31% reduction in pesticide application.

Thailand is a top rice exporter, but its grain yield is low at 3.1 t/ha, compared with other major rice producers like China (6.9 t/ha) and Vietnam (5.5 t/ha) (Suwanmontri et al., 2021). The government has boosted agricultural growth by making pesticides more readily available and affordable. However, weak enforcement of pesticide regulations and the availability of unregistered and counterfeit pesticides make it difficult to control pesticide use and pollution. The rapid expansion of cash crops such as rice, palm oil, and rubber has led to increased pesticide use to protect valuable exports, thereby worsening pollution concerns.

## Need for new toxicological testing methods

More than 140,000 chemicals have been developed for commercial use, and many compounds are present in natural products (Krewski et al., 2020). The intricate interactions between human activities and the environment have spurred the development and production of numerous novel chemicals. Toxicity testing plays a critical role in assessing the safety of these chemicals. Although assessments are conducted in rodents, rabbits, and guinea pigs, their applicability to human health remains a significant concern. Given that these chemicals may possess unknown or unpredictable properties, they require thorough testing to evaluate potential toxicity. Toxicological testing can help us understand how chemicals interact with biological systems and identify their potentially harmful effects. It is especially important for ECs, which are often not fully characterized before their release into the environment. However, it can be expensive and time-consuming. For example, comprehensive testing of a single pesticide can cost up to $20 million and take more than 4 years (Krewski et al., 2020).

Comprehensive testing studies are indispensable for accurately evaluating the potential risks of emerging chemicals in the environment. Conventional testing methods, including acute, sub-chronic, and chronic toxicity tests on animals, are commonly employed. The selection of species for these tests is specific to the type of toxicity being assessed and takes into account factors such as lifespan, availability, and cost. For instance, rats are recommended for subchronic, chronic, carcinogenicity, and reproductive studies, while mice are often preferred for carcinogenicity assessments. These specific tests include reproductive and developmental toxicity testing, as well as carcinogenicity and mutagenicity testing. However, these methodologies may not be sufficient to fully capture the complex interactions between chemicals and biological systems. In response, regulatory bodies have been transitioning towards a more holistic approach. This approach more effectively addresses toxicity by considering the effects of chemical mixtures as a whole, rather than focusing on individual compounds. Novel toxicological testing methods are urgently needed to provide a comprehensive evaluation of the potential hazards posed by ECs, ubiquitous human-made chemicals, and evolving synthetic materials. The rapid pace of technological innovation and chemical production can outpace traditional risk assessment methods, leading to the unintentional release of chemicals with unknown toxicities. The researchers and regulatory bodies must balance the need for accuracy with the need to minimize costs and animal testing.

The field of toxicological assessment is undergoing a major transformation, as scientists are attempting to develop new testing paradigms that can keep pace with the ever-changing landscape of chemical exposure (Ma et al., 2022). The convergence of technological innovation, data-driven science, and computational modeling offers a unique opportunity to revolutionize toxicological testing. High-throughput screening, omics approaches, *in silico* modeling, and *in vitro* assays are promising new methods that can capture complex interactions between chemicals and biological systems, elucidate the mechanisms of toxicity, and predict potential adverse outcomes. By harnessing the power of artificial intelligence (AI) and leveraging large datasets, such innovative methodologies can potentially fill knowledge gaps and rapidly identify hazards posed by chemicals (Perez Santin et al., 2021). These methods emphasize a more predictive and efficient mechanism-based approach reducing the reliance on animal testing to produce greater insighmodeling to test the toxicity oft into the potential health risks associated with chemical exposure.

A study on Southeast Asian edible mussels and clams from the coastal region of Singapore revealed the recovery of active pharmaceutical compounds and (EDCs) using liquid chromatography coupled with tandem mass spectrometry (LC-MS/MS) (Bayen et al., 2015). However, studies focusing on the concentration and toxicity of ECs in organisms exposed to these substances are scarce in the Southeast Asian region. Further research is needed to discuss advanced methods for detecting and analyzing these contaminants, as well as to understand their environmental impact and implications for public health.

### Omics approaches in toxicological assessment

Omics-based toxicological testing is an innovative area of research that plays a vital role in toxicity testing in the 21st century (Krewski et al., 2010) as it allows one to delve at a sub-cellular level in the biological system. The integration of “omics” methodologies, which include genomics, transcriptomics, proteomics, lipidomics, and metabolomics, has revolutionized toxicological assessment. These approaches offer a holistic view of the response of biological systems to chemical exposure, provide a deeper understanding of the complex mechanisms of toxicity and potential risks posed by chemicals, reveal dynamic cellular responses, and pave the way for a more comprehensive understanding of the effects of chemical agents on biological systems. Several studies have investigated the effects of PFAS (Yao et al., 2019; Beale et al., 2022), pharmaceuticals (Collins et al., 2007; Yan et al., 2015), pesticides (Manfei et al., 2019; Goh et al., 2021), and EDCs (Messerlian et al., 2017; Sharma et al., 2022; Maitre et al., 2023).

Genomics, the cornerstone of omics methodologies, is the study of the entire genetic makeup of organisms. This helps in understanding the genetic susceptibility and variations that may influence how individuals respond to chemical exposure. Genomic techniques have been used to identify a wide range of ECs with high sensitivity and specificity and measure their effects on ecosystems and human health. Recent studies have demonstrated the application of genomics in the study of endocrine disruptors (Iguchi et al., 2007; Caballero-Gallardo et al., 2016) and flame retardants (Guan et al., 2016). Meanwhile, transcriptomics studies gene expression and identifies molecular signatures of cellular responses to toxins. By dissecting the orchestration of genes, omics-based approaches have revealed the nuanced pathways and networks that mediate cellular adaptation, stress responses, and potential adverse effects. Proteomics complements these insights by studying proteins that are gene products. Hu et al. (2023) analyzed ECs structures that have the potential to create protein adducts. Metabolomics, the study of small molecules produced and used by cells, can provide a dynamic snapshot of cellular physiology and the changes that occur in response to toxic insults. Metabolites, the end products of omics, help understand biochemical pathways, metabolic disruptions, and potential biomarkers of toxicity. Matich et al. (2019) used metabolomics to explore the ecological consequences of nanoparticles, PPCPs, and pesticides.

The integration of omics approaches into toxicological assessments has ushered in a new era for understanding the impact of chemicals on biological systems. By measuring the entire set of genes, proteins, and metabolites in a cell or tissue, omics methods provide a holistic view of toxicological responses. This information has been used to elucidate molecular mechanisms of toxicity, identify potential biomarkers, and develop more accurate risk assessment methods. An integrated study of genomics, transcriptomics, proteomics, and metabolomics has been conducted to provide a holistic understanding of the cellular responses to ECs (Zhang et al., 2023). Bioinformatics and systems biology play a central role in this process, as they can integrate multidimensional data to create comprehensive models of cellular responses to toxicants. The data generated through omics approaches are large and complex; therefore, advanced computational tools are required to extract meaningful insights.

### *In vitro* models for toxicity testing

As researchers seek to unravel the complex interactions between ECs and biological systems, *in vitro* models provide a vital bridge between traditional toxicology and the contemporary demands of environmental challenges. *In vitro* models allow toxicologists to simulate complex physiological processes and detect toxicological responses that cannot be determined using whole-organism studies. Human cell lines have been used to study the effects of ECs on specific organs and tissues. Solan and Lavado (2022) reviewed toxicity data obtained using human cells *in vitro* and analyzed the advantages and limitations of cell-based models, providing insights into potential solutions to address the challenges that arise with the utilization of these methods in safety assessments. Hoover et al. (2019) used fibroblast cell lines and Quantitative structure–activity relationship modeling to test the toxicity of PFAS. Luis et al. (2016) utilized hemolymph and the subcellular fraction of bivalve *Mytilus galloprovincialis* gold nanoparticles and pharmaceuticals (carbamazepine and fluoxetine).

*In vitro* models have many benefits beyond their ability to elucidate the intricate molecular mechanisms of toxicity. They can be employed to screen large quantities of ECs and assist in the prioritization of chemicals for subsequent assessments. The advent of omics technologies has further enhanced the capabilities of *in vitro* toxicology by determining the global molecular changes induced by ECs (Fang et al., 2020). Zhang et al. (2016) used *in vitro, in vivo,* and *in silico* approaches to study phosphorus-containing flame retardants, and demonstrated that multi-model approaches are of great importance for comprehensively evaluating and accurately predicting the potential health and ecological risks of ECs.

In addition, *in vitro* models can be used to integrate high-throughput data and computational approaches, which can help refine predictive toxicology frameworks. A comprehensive understanding of the intricate network of toxicological interactions between ECs and cellular systems can be achieved using various *in vitro* techniques and methodologies.

## Advancements in toxicity testing in the 21st century

In 2008, the National Toxicology Program (NTP), the National Institute of Health Chemical Genomics Center (NCGC), and the Environmental Protection Agency (EPA) launched the Tox-21 program. This program was launched to advance the development of toxicity tests by leveraging the collective expertise, resources, and tools of each agency, thereby accelerating the prediction of chemical impacts on human health. Tox-21 actively embraces and supports the development and implementation of new approach methodologies (NAMs) within its framework (Krewski et al., 2020). NAMs encompass a broad spectrum of technologies, methodologies, and approaches that provide insights into chemical hazard and risk assessment without relying on animal testing. Utilizing in-chemico, *in silico*, or *in vitro* techniques, these methodologies can generate information that is either comparable to or surpasses that obtained from conventional testing methods (Schmeisser et al., 2023). By closely mimicking human biology and offering mechanistic insights into chemical toxicity, NAMs have the potential to significantly enhance our understanding of chemical hazards and risks to human health.

Advanced cell-based assays use cultured cells and automated systems to rapidly evaluate a wide range of cellular responses. They can provide information on the cytotoxicity, genotoxicity, and endocrine disruption potential of ECs, which can help researchers identify their adverse effects on cellular systems. Organ-on-a-chip models created using microfluidic systems can replicate the physiological microenvironment of different tissues and provide a more precise assessment of toxicological responses (Rothbauer et al., 2021). These innovative approaches provide a comprehensive understanding of how ECs interact with cellular pathways and cause organ-specific toxicities. Although this model is still in its nascent stage, a few research papers have been published on its application to the analysis of environmental contaminants (Lu and Radisic, 2021; Yang et al., 2021).

Human-relevant 3D cell culture models are key tools for the toxicological testing of ECs. These models replicate the physiological microenvironments of human tissues more accurately than traditional 2D cell cultures, providing better predictions of how humans respond to contaminants. These models can reliably assess the toxicological applications of exposure by better mimicking the interactions between contaminants and complex cell systems. Zhang et al. (2022) implemented a metabolomics approach to analyze the comparative profiles of metabolites in 3D human normal liver cell spheroids following PFOA treatment.

The recent synergy between environmental science and AI has led to a paradigm shift, enabling us to address environmental challenges with unprecedented precision and efficiency. Owing to its machine learning algorithms and advanced data analytics capabilities, AI has become a powerful tool for understanding the complexities of ECs. Rodrigues et al. (2021) demonstrated that various distinct ECs can be detected and differentiated by utilizing the photochemical responses of microalgae. Jeong and Choi, (2022) reviewed 96 papers published since 2014 and found that AI models have been developed to predict approximately 30 different toxicity endpoints using more than 20 toxicity databases.

## Future directions and research gaps

Environmental pollution caused by ECs has evolved into a multifaceted problem that requires immediate attention. Given the limited research on the toxicity of mixtures of ECs in Southeast Asia, this review makes an important contribution to the field of environmental toxicology in this region. Diverse sources of ECs, from consumer products to industrial processes, create a complex web of exposure pathways that must be thoroughly investigated. Understanding the dynamics of these contaminants can provide insight into the complex interactions between chemicals and living organisms. This information can be used to make informed decisions regarding environmental management and regulatory frameworks. The diverse physicochemical properties and complex behaviors of ECs in natural systems pose unique challenges to the understanding of their fate, transport, and potential biological effects.

The advent of ECs as a distinct category of environmental pollutants has created a critical juncture for the scientific community, which demands a robust and adaptable approach to toxicological testing. Testing a wide range of chemicals, including ECs, is essential to protect human health and the environment. Existing testing methods may not be sufficiently sensitive for detecting ECs, and new methods must be developed to ensure that these chemicals do not pose a risk to public health. An interdisciplinary approach that combines chemists, biologists, toxicologists, engineers, and informaticians is required to develop new toxicological testing methods. This will help us comprehensively understand chemical safety and effectively manage the risks posed by ECs.
